# Differential Expression Profile of lncRNA in Glioma Cells and the Effect of lncRNA NKX3-1 on Glioma Cells Through Fem1b/SPDEF Pathway

**DOI:** 10.3389/fonc.2021.706863

**Published:** 2021-07-19

**Authors:** Yang Cai, Ming Wang, Yan Cui, Zhigang Tan, Yugang Jiang

**Affiliations:** Department of Neurosurgery, The Second Xiangya Hospital, Central South University, Changsha, China

**Keywords:** lncRNA, NKX3-1, FEM1B, SPDEF, glioma

## Abstract

**Objective:**

To investigate the differential expression of lncRNA in glioma cells, as well as the effect of lncRNA NKX3-1 on glioma cells.

**Methods:**

Glioma-related data were first downloaded from the TCGA database and analyzed using bioinformatics, after which the lncRNA NKX3-1 was chosen for further experiments. The expression of the lncRNA NKX3-1 in glioma tumor samples was detected using qRT-PCR. The subcellular localization of lncRNA NKX3-1 was determined using fluorescence *in situ* hybridization (FISH). CCK-8, flow cytometry, cell scratch, and transwell assays were used to detect cell proliferation, apoptosis, and invasion. The downstream pathway of lncRNA NKX3-1 was investigated using luciferase assays and detected using western blot, transwell, and cell scratch assays.

**Results:**

The differential expression profile of lncRNA in glioma was obtained. NKX3-1 lncRNA was found to be significantly increased in glioma tumor tissues. LncRNA NKX3-1 was found in the nucleus. Proliferation, invasion, and migration of glioma cells were significantly increased (P <0.05) in the lncRNA NKX3-1 overexpression group, while apoptosis ability was significantly decreased (P <0.05). Tumor volume and weight were significantly increased in the lncRNA NKX3-1 overexpression group in nude mice (P <0.05). LncRNA NKX3-1 significantly increased the luciferase activity of Fem1b 3’-UTR-WT reporter genes (P <0.05) as well as the levels of SPDEF protein (P <0.05). The protein level of FEM1B was significantly reduced. Cell invasion and migration were significantly increased (P <0.05) in the lncRNA NKX3-1 overexpression group plus SPDEF group.

**Conclusion:**

We investigated the differential expression profile of lncRNAs in glioma and discovered that the lncRNA NKX3-1 plays an important role in cancer promotion *via* the Fem1b/SPDEF pathway.

## Introduction

Glial cells, as the true companion of neurons, participate in complex processes such as signal transduction and neurotransmission (1). Glioma is the most common primary central nervous system brain tumor, with a relatively high incidence and fatality rate (2). Glioma pathogenesis is caused by both internal and external factors, which refer to genetic susceptibility factors and environmental factors, respectively (3, 4). A number of factors will influence the development and prognosis of gliomas, including several genes, proteins, biomolecules, and interacting environmental factors that will cause gliomas to form (5, 6). If the genetic material has cancer-causing mutations at the cellular level, these reasons can cause the cell to enter the cell cycle mitosis, escape normal apoptosis, and contact inhibition of cell growth, causing the cell to grow. Changes in tumor neovascularization, hypoxia, and necrosis may occur (7, 8). Surgical resection is an option for low-grade non-invasive gliomas. It cannot be completely removed from high-grade gliomas. In most cases, the first choice of treatment method is surgery and adjuvant radiotherapy and chemotherapy. For some recurring gliomas, targeted therapy can also be performed (9).

LncRNA is a long-chain non-coding RNA that regulates the progression and metastasis of certain cancers. NKX3-1 is widely regarded as a highly specific and sensitive marker of prostate adenocarcinoma (10). According to NKX3-1 transcriptome data, its mRNA expression is up-regulated in EWSR1-NFATC2 sarcoma (11). SPDEF is a member of the ETS family and is also known as prostate-derived ETS factor (PDEF) (12). SPDEF was discovered to interact with NKX3-1 and androgen receptor in prostate epithelial cells, regulating the expression of prostate-specific antigen (PSA) (13), but little is known about NKX3-1 through SPDEF-related pathways in glioma. So this study investigates the effect of NKX3-1 on the proliferation, invasion, and migration of glioma cells, as well as the relationship between SPDEF-related pathways.

## Materials and Methods

### Bioinformatic Prediction

Data on glioma-related lncRNA expression were downloaded from the TCGA database and compiled, with 50 normal samples and 371 tumor samples included. The differential lncRNA and mRNA matrices were extracted using segmentation tools, and the format was converted to text character segmentation for heat map drawing and clinical data file merge, as well as survival difference analysis. The databases Gene Ontology (GO) and the Kyoto Encyclopedia of Genes and Genomes (KEGG) were used to examine the functional mechanism pathways.

### Ethics Statement

All patients who took part in the study signed informed consent forms. The Clinical Trial Ethics Committee of The Second Xiangya Hospital approved all experimental procedures.

### Study Subjects

Glioma tissues (n = 20) and paracancerous tissues (n = 20) were collected and stored in liquid nitrogen from glioma patients admitted to our hospital between May 2019 and January 2020. All samples were collected with the consent of the patients and their family members and were approved by our hospital’s ethics committee. Patient information can be seen in **Table 1**. Human normal glial cell (HEB) and glioma cell lines A172, U251, U373, and U87 were obtained from the Chinese Academy of Sciences’ Institute of Biology in Shanghai, China. The Beijing Vitong Lihua Experimental Animal Technology Co., Ltd. provided a 4-week-old female BALB/C-nude mouse weighing 16–18 g. SCXK (Beijing) 2019-0012 is the license number.

**Table 1:** Distribution of characteristics in glioma patients.

Variables	n=20	%
Age (mean±SD)	35.7±10.1	
Gender		
Male	12	60.0
Female	8	40.0
Tumor site		
Frontal lobe	6	30.0
Temporal lobe	7	35.0
Occipital lobe	3	15.0
Parietal lobe	4	20.0

### The Expression of lncRNA NKX3-1 Was Detected by qRT-PCR

Trypsin (Hyclone, America) was used to digest glioma tissue, paracancerous tissue, human normal glial cells (HEB), and glioma cell lines A172, U251, U373, and U87. The RNA was extracted using an RNA kit (Promega Corporation, USA), the RNA concentration was determined, and the RNA was reverse transcribed into cDNA. For the qRT-PCR reaction, SYBR Premixture Ex TaqII (Thermo Fisher Scientific, USA) was used. The lncRNA NKX3-1 upstream sequence was 5’-CCCACACTCAGGTGATCGA-3’, and the downstream sequence was 5’-GAGCTGCTTTCGCTTAGTCTT-3’. The internal reference was -actin, and the primers were upstream 5’-GCTAATATCTATAATC-3’ and downstream 5’-GAGGCTATCTTCATAGAT-3’. The sequence of lncRNA NKX3-1 is available in the supplementary material. The reaction temperatures were 92°C for 20 min, 90°C for 10 min, and 82°C for 10 min and amplified by 40 cycles.

### Fluorescence *In Situ* Hybridization Was Used to Determine the Subcellular Localization of lncRNA NKX3-1

The cell slide (cover glass) was fixed with 4% paraformaldehyde, and the fixed solution was washed away with PBS. The following operations were carried out in order: Glycine treatment for 5 min, followed by PBS washing; 0.4% TritonX-100 treatment for 15 min, followed by PBS rinse; and protease treatment for 15 min, followed by PBS rinse. Fixed with 4% paraformaldehyde and washed with PBS; 0.25% acetic acid was treated with alcohol for 10 min, washed with PBS, and dried. After placing the cover glass on the slide, the hybridization solution was added for 1 h of pretreatment. After incubating for 16 h with a biotin-labeled p lncRNA NKX3-1 probe hybrid, an alkaline phosphatase-labeled anti-biotin antibody (1:1,000 dilution) was added for further hybridization. Secondary antibody with fluorescence labeling (diluted 1:2,000) was added, and the nuclei were stained with DAPI for examination under a fluorescence microscope.

### Cell Culture, Transfection, and Grouping

The U87 cell line was cultured in DMEM medium (Hyclone, America) in a 5% CO2 incubator at 37°C. When the cell fusion reached 90%, the cells were washed with PBS, digested with 0.25% trypsin, and formed into a single-cell suspension. The cells were passed every two days, and the logarithmic growth phase cells were chosen for the following experiments. Cells from the logarithmic growth phase were collected and inoculated into a 12-well plate. The plate was laid 24 h after transfection. When the cells had grown to 80% the next day, Lipofectamine 3000 transfection reagent (Thermo Fisher Scientific, America) was used and transfected at a concentration of 40 nmol/L per cell, as directed. They were then divided into three groups: the control group (which received no transfection), the NKX3-1 overexpression group (which was transfected with pcDNA-lncRNA-NKX3-1) and the NKX3-1 knockdown group (transfected with si-lncRNA-NKX3-1). Follow-up experiments were performed after shaking the culture plates and incubating them for 24 h in an incubator at 37°C and 5% CO2.

### Cell Proliferation Was Detected by CCK-8

Cell proliferation was detected using the CCK-8 kit (Thermo Fisher Scientific, America). Each group’s transfected cells were inoculated in 96-well plates and incubated at 37°C for 24, 48, 72, and 96 h. CCK-8 reagent was added and incubated for another 2 h before the absorbance value was measured with a 490 nm spectrophotometer.

### Cell Apoptosis Was Detected by Flow Cytometry

After intervention, three groups of U87 cell lines were chosen and inoculated into 24-well plates with 2,104 cells per well. After an overnight incubation, the Annexin V-FITC-PI Apoptosis Kit (Thermo Fisher Scientific, America) was stained according to the manufacturer’s instructions and flow cytometry was used to analyze the results. All of the experiments were carried out three times.

### Cell Invasion Was Detected by the Transwell Assay

After three intervention groups, 5 × 10^4^ U87 cell lines were suspended in serum-free DMEM medium and placed in a transwell chamber with basement membrane matrix coating. The cells were then cultured in 500 μl of culture medium containing 10% FBS for 48 h in an incubator at 37°C and 5% CO2. Non-invasive cells were removed with a cotton swab after cells that had penetrated the membrane were fixed with 100% methanol and stained with 0.1% crystal violet. Under a microscope, the stained cells are imaged, and the cells that invade the bottom surface are counted.

### Cell Scratch Test

Following intervention, three groups of U87 cell lines were chosen and inoculated in a 6-well plate. When the cells had reached 90% fusion, scratches were made in the cell monolayer with the tip of a sterile straw (100 l), the cells were washed with PBS, and cultured in serum-free medium. The relative distance of cell migration was measured after 24 h using a low-power phase contrast microscope, and the migration rate of the scratches was calculated.

### Tumor Growth in Nude Mice

Twenty 4-week-old female BALB/C-nude mice were randomly assigned to one of two groups: no-load and NKX3-1 overexpression. Subcutaneous injections of 0.15 ml of pcDNA3.1 NC cell suspension transfected with pcDNA-lincRNA-NKX3-1 cell suspension transfected with NKX3-1 overexpression were given to the right no-load group. Every three days after solid tumors were discovered, the length and short diameters of the tumors were measured with calipers. The tumor volume was determined using the formula V = (Length × Width2)/2. The nude mice were killed 35 days after inoculation, and the tumor bodies were separated and weighed.

### Double Luciferase Reporter System Experiment

The TargetScan website predicted the potential binding sites of lncRNA NKX3-1 at the 3’-UTR of Fem1b mRNA. The 3’-UTR sequence of the lncRNA NKX3-1 binding wild-type (WT) or mutated (MUT) was amplified from the genome and cloned into the PGL-3 luciferase reporter vector. The luciferase reporter vector was co-transfected into cells from both the NKX3-1 overexpression and control groups. The luciferase activity was detected by a double luciferase reporter test box after 48 hours, and the relative luciferase activity of the firefly was calculated.

### Protein Expression Was Detected by Western Blot

Total proteins were isolated from the tissues of nude mice in the no-load and NKX3-1 overexpression groups, and protein concentrations were determined using a BCA protein detection kit. The proteins were electrophoresed, migrated to PVDF membrane, sealed with skimmed milk, and incubated overnight at 4°C with FEM1B (1:1,000 dilution), SPDEF (1:1,000 dilution), ROCK1 (1:1,000 dilution), c-Myc (1:500 dilution), Akt (1:500 dilution), primary antibody (1:1,000 dilution), and -actin. SPDEF protein levels were measured with an enhanced chemiluminescence detection system. Antibodies were obtained from Thermo Inc. in the United States.

### Rescue Experiment

U87 cell lines were divided into five groups: control, NKX3-1 overexpression, NKX3-1 overexpression +SPDEF blank, NKX3-1 overexpression +SPDEF, and NKX3-1 knockdown. The control group’s cells were not transfected. The NKX3-1 overexpression group received pcDNA-lincRNA-NKX3-1, while the NKX3-1 overexpression group +SPDEF blank received pcDNA-lincRNA-NKX3-1 and SPDEF NC. The NKX3-1 overexpression group was transfected with CAT.ORB13642 and pcDNA-lincRNA-NKX3-1, while the NKX3-1 knockdown group was transfected with si-lincRNA-NKX3-1. Cell scratch and invasion experiments were carried out 48 h after transfection.

### Statistical Analysis

Processing was carried out using the SPSS23.0 software. The chi-square test was used to analyze the count data, which was expressed as a percentage. The t test was used for pair comparisons. The difference of P <0.05 was statistically significant.

## Results

### Screening of Differentially Expressed lncRNAs

**Figure 1:**
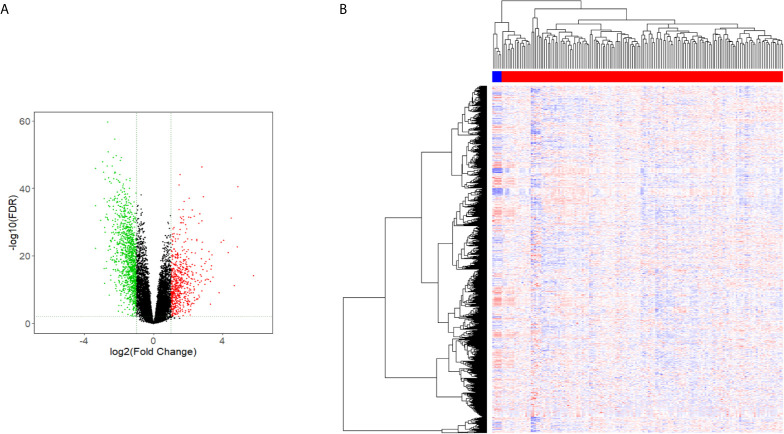
Volcano and heat maps of differential gene expression. **(A)** Volcano Plot of lncRNAs expression in glioma. **(B)** Heat map of lncRNAs expression in glioma.

The Limma package analysis results were derived, and the volcano map preliminarily showed the expression of differential genes, as shown in **Figure 1A**, and the heat map showed the expression of differential genes, as shown in **Figure 1B**. The information for the 20 genes with the greatest expression differences can be found in **Table 2**.

**Table 2:** Differential expression of lncRNA in glioma tissues.

LncRNA (up-regulated)	logFC	P Value	FDR	LncRNA (down-regulated)	logFC	P Value	FDR
PCA3	4.679379	9.54E−13	6.05E−12	ADAMTS9-AS1	−2.56539	1.99E−39	4.70E−37
AP006748.1	4.062486	7.03E−27	2.58E−25	PGM5-AS1	−2.48591	9.64E−27	3.47E−25
LINC02170	3.307527	6.26E−07	1.89E−06	AL049555.1	−2.35735	1.53E−27	6.18E−26
PCAT14	3.105758	9.81E−15	8.07E−14	AC005180.1	−2.23164	1.47E−33	1.33E−31
AC009119.1	3.028928	3.61E−20	5.69E−19	MIR205HG	−2.20447	5.59E−13	3.65E−12
AP004608.1	3.001164	1.77E−14	1.41E−13	AC005180.2	−2.15984	2.88E−36	4.03E−34
AP000696.1	2.826104	2.29E−26	7.82E−25	LINC01018	−1.98815	7.96E−26	2.44E−24
AC092535.4	2.811437	4.95E−11	2.55E−10	GAS1RR	−1.98644	1.20E−34	1.36E−32
PCAT7	2.793721	5.59E−33	4.75E−31	AP000808.1	−1.92869	8.49E−16	8.00E−15
AC144450.1	2.784229	1.13E−15	1.05E−14	AF165147.1	−1.91284	5.88E−26	1.86E−24
AC141930.1	2.705916	4.21E−16	4.08E−15	AP001107.5	−1.88507	7.17E−25	2.02E−23
ERVH48-1	2.660447	3.33E−09	1.36E−08	LINC00844	−1.85879	1.03E−15	9.64E−15
PCAT29	2.615271	3.07E−32	2.38E−30	ADAMTS9-AS2	−1.82886	6.90E−35	8.13E−33
AC104667.2	2.503502	3.68E−27	1.39E−25	SNHG18	−1.81496	2.24E−28	1.00E−26
PCAT1	2.487096	1.28E−16	1.31E−15	C20orf166-AS1	−1.72467	1.52E−19	2.26E−18
DRAIC	2.386532	4.59E−19	6.50E−18	LINC02562	−1.67517	6.85E−09	2.70E−08
AL031123.2	2.3554	1.30E−34	1.47E−32	LINC01679	−1.65862	6.38E−28	2.72E−26
AL589182.1	2.291948	2.11E−10	1.00E−09	FGF14-AS2	−1.59352	1.86E−23	4.36E−22
FOXP4-AS1	2.254677	1.74E−36	2.52E−34	AC036108.3	−1.57101	3.59E−26	1.18E−24
AC100826.1	2.245624	3.57E−07	1.11E−06	MAGI2-AS3	−1.55998	3.15E−40	8.58E−38

### Batch Survival Analysis

**Figure 2:**
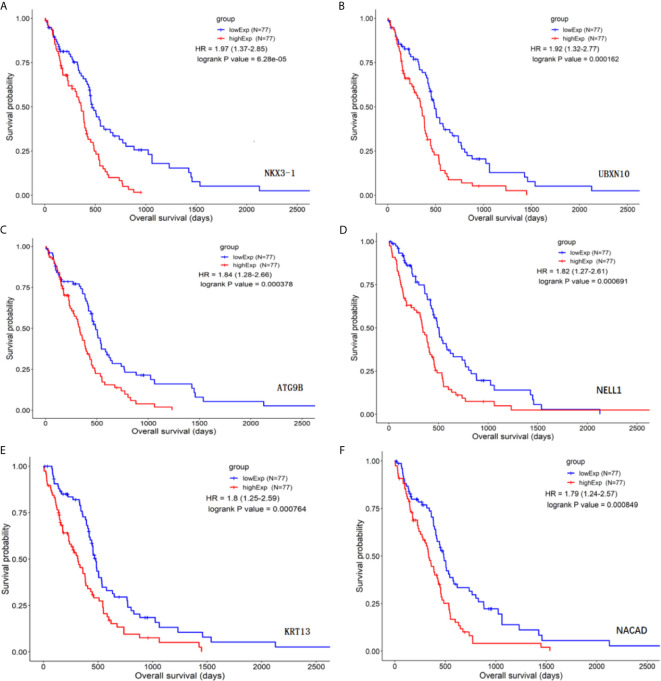
Survival curve analysis of differential genes (top six genes by P value). **(A)** The survival curve for NKX3-1. **(B)** The survival curve for UBXN10. **(C)** The survival curve for ATG9B. **(D)** The survival curve for NELL1. **(E)** The survival curve for KRT13. **(F)** The survival curve for NACAD.

The differential genes listed above were used for survival differential analysis, sorted by P value, and survival curves of the top six differentially expressed lncRNAs with the lowest P value were chosen, as shown in **Figure 2**. Significant differences in NKX3-1 survival curves were discovered, so one of the lncRNA NKX3-1 that was differentially expressed and had a significant effect on survival was chosen for further research.

### Results of GO and KEGG Enrichment Analysis

**Figure 3:**
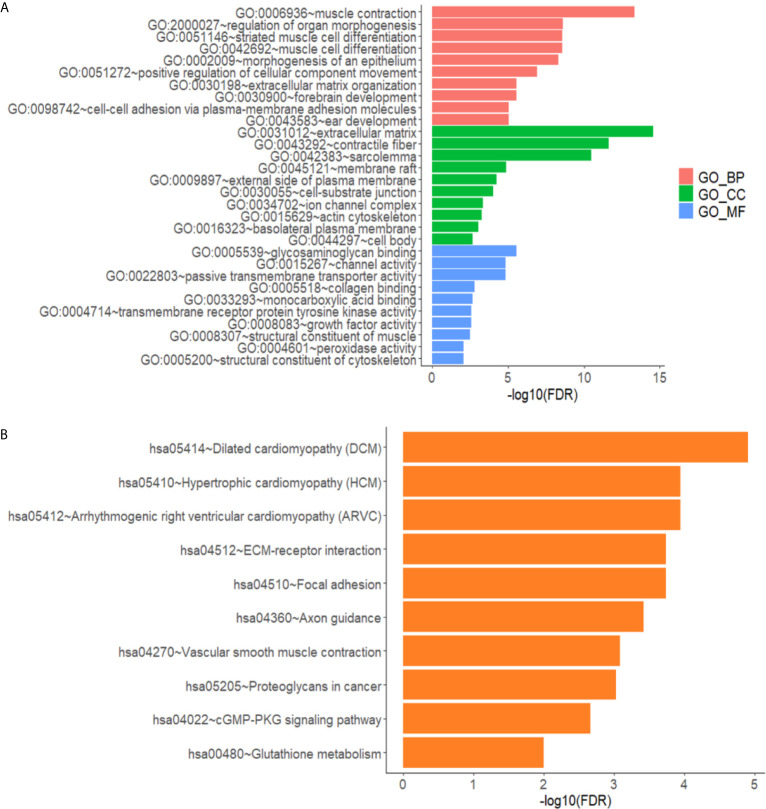
Annotated statistical chart of Go and KEGG analysis. **(A)** GO analysis results of lncRNAs in glioma. **(B)** KEGG analysis results of lncRNAs in glioma.

The differentially expressed genes in the gene ontology analysis were related to muscle contraction, positive regulation of cellular component movement, extracellular matrix, and so on, according to GO analysis, as illustrated in **Figure 3A**. As shown in **Figure 3B**, the KEGG analysis focused on cytokine–cytokine receptor interactions, transcriptional error regulation in cancer, and other related pathways.

### Expression of NKX3-1 in Glioma Carcinoma, Adjacent Tissues and Glioma Cells

**Figure 4:**
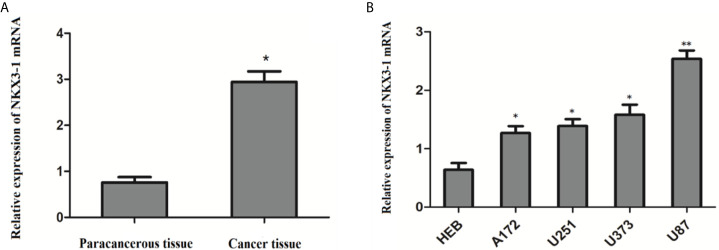
Relative expression of lncRNA NKX3-1 in tissues and cells. **(A)** Expression of NKX3-1 mRNA in glioma and paracancerous tissues. *P < 0.05. **(B)** Expression of NKX3-1 mRNA in different cells. *P < 0.05, **P < 0.01 (compared with HEB cell).

QRT-PCR results showed that the relative expression of lncRNA NKX3-1 was significantly increased in glioma tissues compared with paracancerous tissues (P <0.05). The relative expression of lncRNA NKX3-1 in glioma cell lines A172, U251, U373 and U87 was significantly higher than that in HEB cell lines (P <0.05), and the relative expression of lncRNA NKX3-1 in U87 cell line was the highest compared with that in HEB cell line (P <0.001), as shown in **Figure 4**. Therefore, U87 cell line was selected for subsequent experiments in the later stage.

### Localization of lncRNA NKX3-1 in Glioma Cells

**Figure 5:**
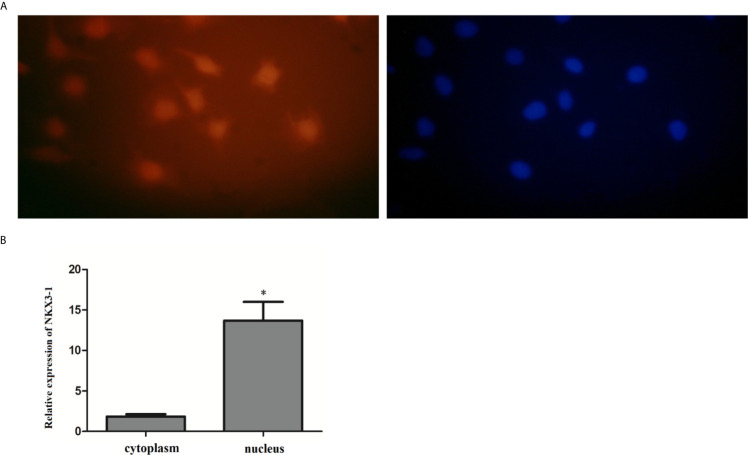
Expression of lncRNA NKX3-1 in the cytoplasm and nucleus of glioma cell. **(A, B)** Results of fluorescence *in situ* hybridization. *P < 0.05.

The FISH assay results revealed that NKX3-1 was mostly found in the nucleus of glioma cells, but a small amount was also found in the cytoplasm. The expression of NKX3-1 in the nucleus was (13.69 ± 1.73%) after nucleoplasmic separation and qRT-PCR. As shown in **Figure 5**, it was significantly higher than that in cytoplasm (1.84 ± 0.53%) (P <0.05).

### Effect of lncRNA NKX3-1 on Proliferation and Apoptosis of Glioma Cells

**Figure 6:**
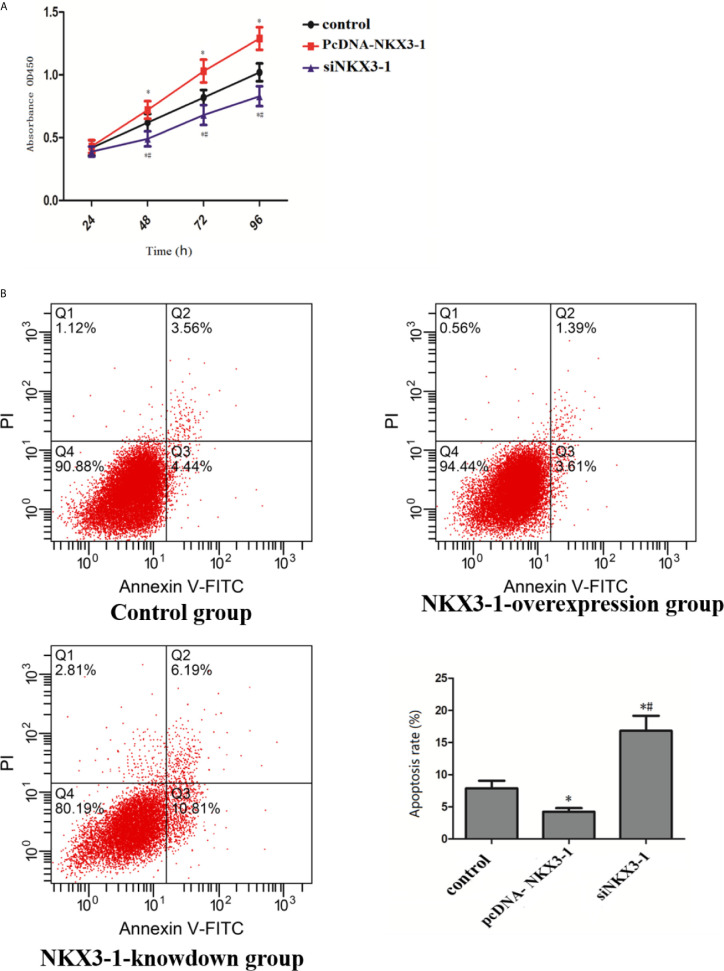
Effect of lncRNA NKX3-1 on proliferation and apoptosis of glioma cells. **(A)** CCK8 results of control, PcDNA-NKX3-1 and siNKX3-1 group. **(B)** Apoptosis assay results of control, PcDNA-NKX3-1 and siNKX3-1 group. *P < 0.05 (compared control with pcDNA-NKX3-1), ^#^P < 0.05 (compared pcDNA-NKX3-1 with siNKX3-1).

CCK assay results showed that, when compared to the control group, the lncRNA NKX3-1 overexpression group significantly increased the proliferation ability of glioma cells at 48, 72, and 96 h (P <0.05), whereas the proliferation ability of glioma cells in the lncRNA NKX3-1 knockdown group significantly decreased at 48, 72, and 96 h (P <0.05), as shown in **Figure 6A**. As shown in **Figure 6B**, lncRNA NKX3-1 overexpression significantly reduced the apoptotic ability of glioma cells compared to the control group (P <0.05), whereas lncRNA NKX3-1 knockdown significantly increased the apoptotic ability of glioma cells (P <0.05).

### Effect of lncRNA NKX3-1 on Invasion and Migration of Glioma Cells

**Figure 7:**
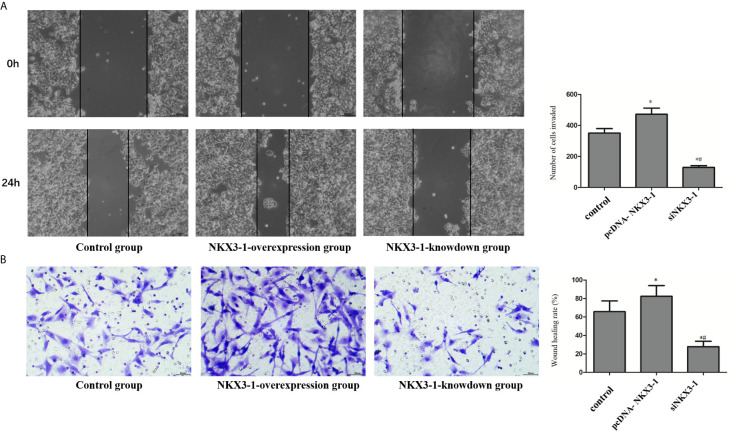
Effect of lncRNA NKX3-1 on invasion and migration of glioma cells. **(A)** Cell scratch test results of control, PcDNA-NKX3-1 and siNKX3-1 group. **(B)** Transwell assay results of control, PcDNA-NKX3-1 and siNKX3-1 group. *P < 0.05 (compared control with pcDNA-NKX3-1), ^#^P < 0.05 (compared pcDNA-NKX3-1 with siNKX3-1).

As shown in **Figure 7A**, the invasion ability of glioma cells in the lncRNA NKX3-1 overexpression group was significantly higher than that in the control group (P <0.05), whereas the invasion ability of glioma cells in the lncRNA NKX3-1 knockdown group was significantly lower than that in the control group and lncRNA NKX3-1 overexpression group (P <0.05). As shown in **Figure 7B**, the wound healing rate of glioma cells in the lncRNA NKX3-1 overexpression group was significantly higher than that of the control group (P <0.05). The wound healing rate of glioma cells in the lncRNA NKX3-1 knockdown group was significantly lower than that of the control group and the lncRNA NKX3-1 overexpression group (P <0.05).

### Effect of lncRNA NKX3-1 on Tumor Growth in Nude Mice

**Figure 8:**
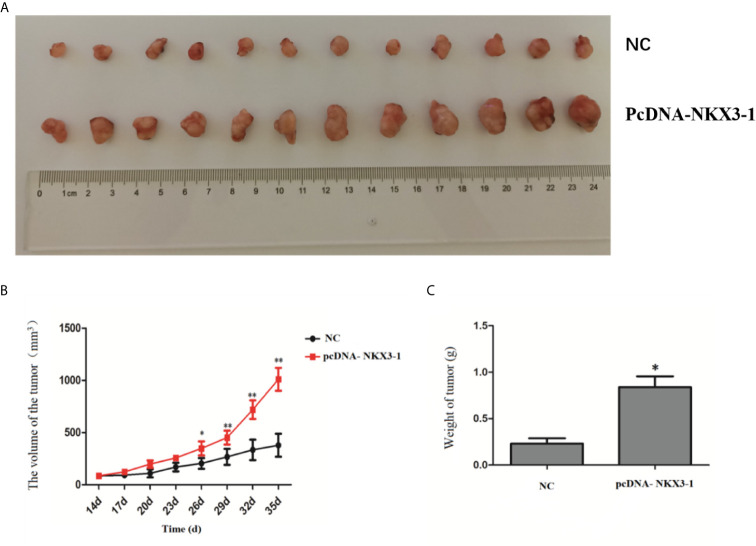
Effect of lncRNA NKX3-1 on tumor growth in nude mice. **(A)** Result of tumors taken from tumor-bearing mice. **(B)** Tumor volume growth curves in NC and pcDNA-NKX3-1 groups. **(C)** Weight tumor in NC and pcDNA-NKX3-1 groups. *P < 0.05, **P < 0.01.

**Figure 8** shows that tumor volume and weight were significantly higher in the lncRNA NKX3-1 overexpression group compared to the control group (P <0.05).

### LncRNA NKX3-1 Targets Expression of Fem1b

**Figure 9:**
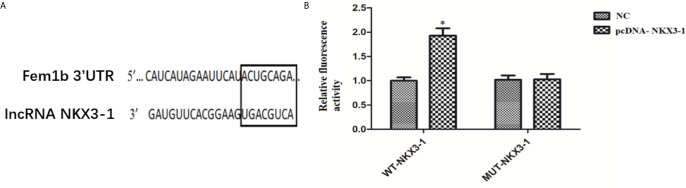
Results of double luciferase report assay. **(A)** The binding site of lncRNA NKX3-1 and Fem1b. **(B)** Relative fluorescence activity in NC and pcDNA-NKX3-1 group. *P < 0.05.

The TargetScan website predicted the potential target genes of NKX3-1, and the 3’-UTR region of Fem1b was discovered to have the targeted binding site of NKX3-1. As shown in **Figure 9**, the dual luciferase reporter assay revealed that NKX3-1 significantly increased luciferase activity of the Fem1b 3’-UTR-WT reporter gene compared to the control group (P <0.05), but had no effect on the activity of the Fem1b 3’-UTR-MUT reporter gene (P <0.05).

### Effect of Overexpression of lncRNA NKX3-1 on Tumor-Related Proteins in Nude Mice

**Figure 10:**
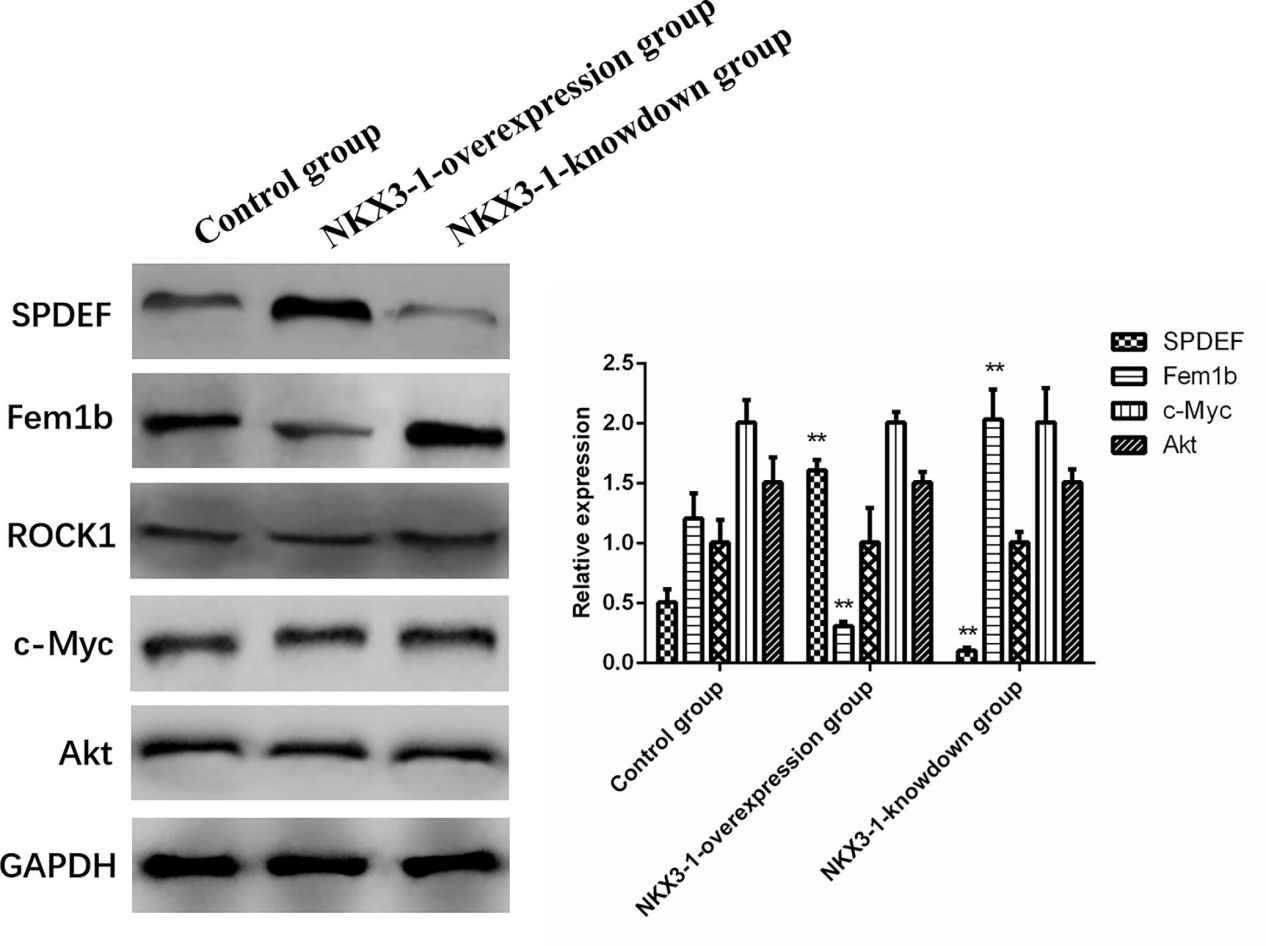
Effects of overexpression and knockdown of lncRNA NKX3-1 on expression of related proteins. **P < 0.01 (compared with control group).

SPDEF protein levels were significantly higher in the lncRNA NKX3-1 overexpression group compared to the control group (P <0.05), while Fem1b protein expression was significantly lower in the lncRNA NKX3-1 knockdown group, while the opposite was true in the lncRNA NKX3-1 knockdown group. **Figure 10** shows that there were no significant differences in the protein levels of ROCK1, c-Myc, and Akt in the other three groups (P >0.05).

### The Effect of lncRNA NKX3-1 on Invasion and Migration of Glioma Cells by Up-Regulating SPDEF

**Figure 11:**
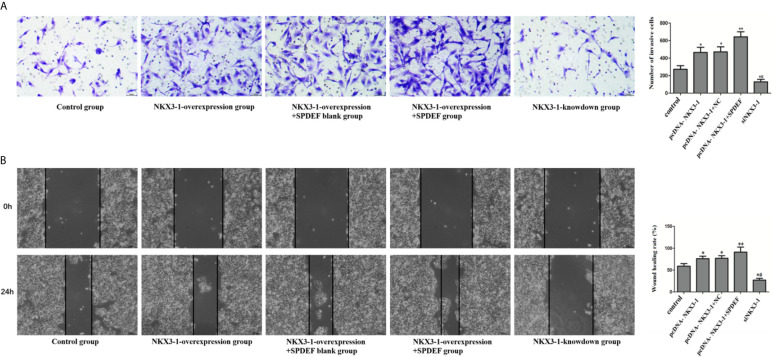
The effect of NKX3-1 on invasion and migration of glioma cells by up-regulating SPDEF. **(A)** Transwell assay results of control, NKX3-1 overexpression, NKX3-1 overexpression +SPDEF blank, NKX3-1 overexpression +SPDEF and NKX3-1 knockdown groups. **(B)** Cell scratch test results of control, NKX3-1 overexpression, NKX3-1 overexpression +SPDEF blank, NKX3-1 overexpression +SPDEF and NKX3-1 knockdown groups. * P<0.05, **P<0.01 (compared with control group), ^#^P<0.05 (compared pcDNA-NKX3-1 with siNKX3-1).

Compared with the control group, cell invasion and migration were significantly enhanced in the NKX3-1 overexpression group and the NKX3-1 overexpression group +SPDEF blank group (P <0.05), compared with NKX3-1 overexpression group, cell invasion and migration ability in NKX3-1 overexpression group +SPDEF group was significantly increased (P <0.05), the invasion and migration of NKX3-1 knockdown group was significantly lower than that of the control group (P <0.05), as shown in **Figure 11**.

## Discussion

Glioma is the most common intracranial malignant primary brain tumor, accounting for approximately 80% of all brain malignant tumors. It is highly malignant, has invasive growth, is difficult to remove, and recurs frequently after resection (11). Only 2% of proteins in the human genome are transcribed from coding genes, while the remaining 90% of eukaryotic genome DNA transcription products are made of non-coding RNA, including lncRNA, which can participate in the genome at various levels. The regulation process of lncRNA, such as epigenetics, transcription level, and post-transcription level, has been studied, but the mechanism of action of lncRNA has not been studied (14).

According to relevant research findings, 1ncRNA is closely related to the occurrence and progression of cancer (15). These lncRNAs can interact with DNA, RNA, protein molecules, or their combinations to regulate chromatin organization, transcription, and post-transcriptional regulation (16). The ability of tumor initiation, growth, and metastasis is affected by abnormal lncRNA expression (17). Among them, lncRNA can function in tumor tissues as both a proto-oncogene and a tumor suppressor gene, and it can be used as an important reference index for tumor diagnosis and prognosis (18).

This study looked at the differential expression of lncRNA in glioma cells as well as the effect of LncRNA NKX3-1 on glioma cells *via* SPDEF-related pathways. As a result, we were able to obtain a profile of lncRNA differential expression in glioma cells. After analyzing the differences in survival, the results revealed that lncRNA NKX3-1 was differentially expressed in the glia and had a significant impact on survival, so lncRNA NKX3-1 was chosen for further investigation.

The results of qRT-PCR detection revealed that the relative expression of lncRNA NKX3-1 in glioma tissues was significantly higher when compared to adjacent tissues. LncRNA NKX3 was found in the glioma cell lines A172, U251, U373, and U87. The relative expression of −1 in U87 cells was significantly higher than in HEB cells, and the relative expression of lncRNA NKX3-1 in U87 cells was the highest when compared to HEB cells. As a result, the U87 cell line was chosen for further testing at a later stage. The FISH method revealed that the lncRNA NKX3-1 was mostly found in the nucleus of glioma cells, with only a small amount found in the cytoplasm. The proliferation, apoptosis, invasion, and migration abilities of glioma cells were tested to confirm the function of lncRNA NKX3-1. The findings demonstrated that glioma cell proliferation, invasion, and migration can be aided by the overexpression of the lncRNA NKX3-1. Glioma cells’ apoptotic characteristics should be reduced. Following that, we continued to conduct *in vivo* experiments for verification, and the results showed that tumor volume and tumor weight increased significantly in the lncRNA NKX3-1 overexpression group. Using the TargetScan website to predict potential NKX3-1 target genes, it was discovered that NKX3-1 significantly increased the luciferase activity of the Fem1b 3’-UTR-WT reporter gene. Furthermore, NKX3-1 and Fem1b protein levels have a negative correlation, whereas SPDEF protein levels have a positive correlation. To validate the correlation between NKX3-1 and SPDEF, cell invasion and migration ability in the NKX3-1 overexpression group + SPDEF group was significantly higher than in the NKX3-1 overexpression group. LncRNA plays a role in glioma cell proliferation, either promoting or inhibiting it. Current research indicates that a number of lncRNAs, including lncRNA PLAC2 (19), lncRNA KTN1AS1 (20), lncRNA DANCR (21), lncRNA MIR31HG (22), and others, regulate the proliferation of glioma cells *via* various signaling mechanisms. Overexpression of lncRNA ZEB1-AS1 has been shown *in vitro* to promote the proliferation, migration, and invasion of glioma cells, as well as the cell cycle, so lncRNA ZEB1-AS1 is used as a proto-oncogene in glioma tissues (23). Similar to this study, previous research has shown that SPDEF has a positive regulatory effect on the proliferation of gastric cancer cells (24).

Finally, the lncRNA NKX3-1 is expressed differently in glioma and has a significant effect on survival. Overexpression of the lncRNA NKX3-1 can promote glioma cell proliferation, invasion, migration, and growth while inhibiting apoptosis. The mechanism may be to promote SPDEF expression by inhibiting FEM1B expression. In order to fulfill its role in cancer promotion. In the future, we hope to further study the mechanism related to lncRNA NKX3-1, and introduce new technologies such as nanoparticles to study it from different perspectives.

## Data Availability Statement

The original contributions presented in the study are included in the article/**Supplementary Material**. Further inquiries can be directed to the corresponding author.

## Ethics Statement

The studies involving human participants were reviewed and approved by Clinical Trial Ethics Committee of The Second Xiangya Hospital. The patients/participants provided their written informed consent to participate in this study. The animal study was reviewed and approved by Clinical Trial Ethics Committee of The Second Xiangya Hospital. Written informed consent was obtained from the owners for the participation of their animals in this study.

## Author Contributions

YCa wrote the manuscript. YCa, MW, and YCu performed the experiments together. YJ designed the experiments and performed the data analysis. All authors contributed to the article and approved the submitted version.

## Funding

This study is supported by Hunan Provincial Key Research and Development Program of Science and Technology Innovation Plan (grant no:2018SK21213). This study is also supported by Hunan Provincial Youth Science Fund (grant no:2019JJ50873).

## Conflict of Interest

The authors declare that the research was conducted in the absence of any commercial or financial relationships that could be construed as a potential conflict of interest.
