# Supplementary figures and images for: Differential Expression Profile of lncRNA in Glioma Cells and the Effect of lncRNA NKX3-1 on Glioma Cells Through Fem1b/SPDEF Pathway
Source: Front Oncol. 2021 Jul 19;11:706863. doi: 10.3389/fonc.2021.706863 (PMC8328487; doi:10.3389/fonc.2021.706863)

**Supplementary Figure 1 Sequence information of lncRNA NKX3-1**


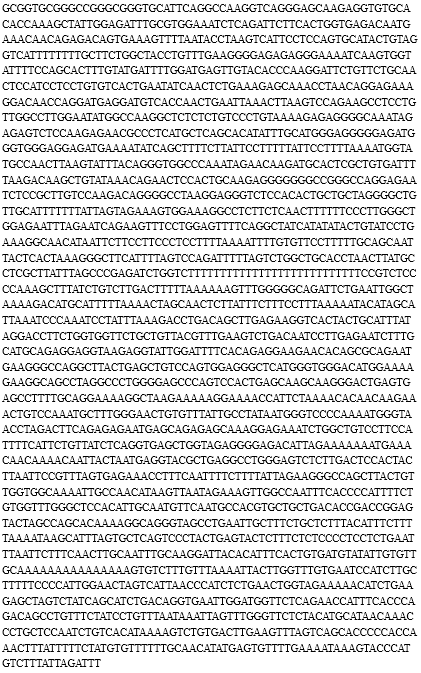

Supplement: Supplementary file 1 [file DataSheet_1.docx]
